# An Arduino-Powered Device for the Study of White Perception beyond the Visual Chromatic Critical Flicker Fusion Frequency

**DOI:** 10.3390/jimaging10070163

**Published:** 2024-07-10

**Authors:** Francisco J. Ávila

**Affiliations:** Departamento de Física Aplicada, Facultad de Ciencias, Universidad de Zaragoza, 50009 Zaragoza, Spain; avila@unizar.es

**Keywords:** Arduino, critical flicker frequency fusion, temporal vision, high-order aberrations, color vision, photo-stress recovery time

## Abstract

Arduino microcontrollers are used for a wide range of technological and biomedical applications, such as image classification, computer vision, brain–computer interaction and vision experiments. Here, we present a new cost-effective mini-device based on RGB LED flicker stimulation for the assessment of the chromatic temporal resolution of the visual function based on the concept of critical flicker fusion frequency (CFF). The assembly of the device and its testing in thirty young subjects demonstrate the steady white visual perception of a trichromatic flicker stimulus (mixture of red, green and blue stimuli) beyond the CFF. Macular function as measured by photo-stress recovery time (PRT) was found to be independent of the CFF measurements for red, green and blue lights. However, a statistical correlation was found between the contrast modulation for CFF for red and green stimuli and PRT. Finally, wavefront measurements demonstrate that high-order aberrations improve the temporal resolution of the visual function.

## 1. Introduction

Visual Acuity is a widespread test to evaluate visual function [1]. However, contrast sensitivity, stereopsis, color perception and temporal vision are also important factors of visual function that are frequently relegated in clinical practice [2]. The temporal aspects of vision are mainly visual latency [3], perisaccadic compensation, persistence [4], temporal integration [5] and resolution [6].

Time perception is also influenced by non-temporal aspects such as the contrast and orientation of the visual test [7]. Visual persistence can be understood as prolonged visual perception for a short time after the physical stimuli are removed. From the point of view of retinal physiology, visual persistence is due to light-adaptive gain control mechanisms in the response of ganglion cells [8].

The perception of objects in motion requires the integration of spatiotemporal information [9]. In this sense, the temporal integration window (TIW) is the period within which separate stimuli will be perceived as a single stimulus. Stimuli presented in sequence within a TIW of around 40 milliseconds are integrated into a single visual perception. Larger TIWs allow for temporal resolution and then the subjective perceptual experience of motion [10]. Cortical visual impairment [11] (CVI) affects processing functions in the temporal, parietal and frontal lobes of the brain; one of the CVI characteristics is the slow visual response when a visual target is presented; and this delayed response is called visual latency and could require suprathreshold stimuli to achieve a visual response [12].

The common way to measure the temporal resolution of visual performance is the critical flicker frequency fusion (CFF), which is the measurement of the temporal frequency of a periodically modulated flickering light at which the stimuli cannot be distinguished at any modulation amplitude [13].

Its experimental simplicity has made the CFF the most evaluated representative temporal aspect of the visual function. CFF has been reported as a potential functional measure of temporal vision in multiple sclerosis [14,15,16], retrobulbar neuritis [17], demyelinating optic neuritis [18] and cognitive performance [19,20].

Spatial resolution and color vision are mediated by cone photoreceptors that encode information for blue–yellow (B/Y) and red–green (R/G) channels and luminance through different cortical mechanisms [21,22]. Furthermore, the R/G and B/Y components combine for the spatiotemporal modulation of color vision [23]. In this sense, patients with degenerative retinopathy suffer from color vision impairment that can be examined psychophysically with chromatic CFF tests. Gregori et al. [24] found altered CFF values for red light in patients with optic neuritis and impaired CCF for blue stimuli in patients with diabetic retinopathy.

In addition, the trichromatic (red, green and yellow) CFF was proposed as a visual test to discriminate between patients with a cataract who are with and without macular affection [25]. The finding revealed a greater sensitivity in yellow CFF to identify macular diseases.

Prior to this work, an Arduino-based LED stimulation device was reported for cognitive research in rodent models and heterochromatic flicker photometry in humans [26]. Taking into account the relationship between retinal and post-retinal vision disorders and color vision impairment, the objective of this work is to present a new configuration of a portable and cost-effective Arduino-powered device for the assessment of the chromatic critical flicker frequency fusion. The device allows the subjective evaluation of the CFF of almost any possible RGB combination and has been tested in 30 young adult volunteers. A new experimental psychophysical phenomenon related to the Talbot–Plateau law is observed: at a given flicker frequency in sequential sampling of red, green and blue colors, a continuous white light stimulus is perceived. In addition, ocular wavefront measurements revealed that high-order aberrations improve the temporal resolution of vision.

## 2. Materials and Methods

### 2.1. Chromatic Critical Flicker Fusion Frequency Device

**Figure 1:**
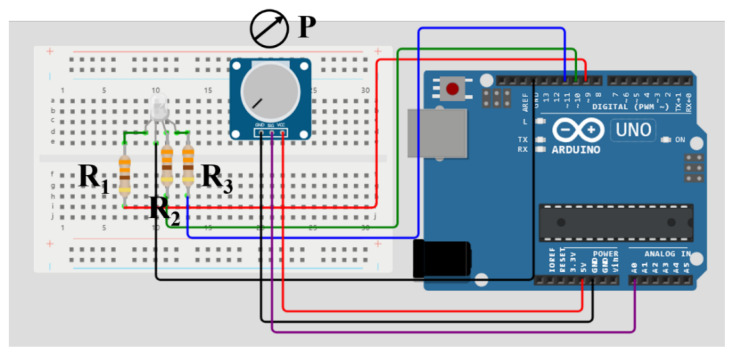
Circuit design for the trichromatic critical flicker frequency fusion device. R_1_, R_2_ and R_3_ are 330 Ω resistors and P a tunable potentiometer.

The chromatic critical flicker fusion frequency (cCFF) device consists of a flickering visual stimulus driven by an open-source USB programmable 32-bit microprocessor (Arduino UNO, arduino.cc). An RGB LED (L-154A4SURKQBDZGW, Kingbright) with 465/525/630 nm predominant wavelengths is controlled by pulse-width modulation (PWM) signals through the Arduino controller. Figure 1 shows the design of the tCFF device, and the red/green/blue cathodes are connected through three resistors (R_1_–R_3_, 330 Ω) to the Arduino digital pins with PWM capability (in this case, −9, −10 and −11 for channels red, green and blue, respectively). While the possible combination of the three RGB channels (expressed from 0 to 255 saturation values, i.e., 100% contrast modulation) allows for the generation of 16.777.216 colors, the experiment focused on the flickering of three monochromatic stimuli for 465 (blue), 525 (green) and 630 (red) nm RGB channels.

The Arduino code (Sketch) to control the cCFF device is shown in Table 1. The Integer “sensorValue” reads a value from the potentiometer (P) connected to the analog in (A0); then, the function “delay ()” controls the pause length of the “sensorValue” value in milliseconds. Finally, the “Serial.printl ()” command prints the flicker rate data (in ms). The luminance modulation of the RGB LED was set between 0 and 255 (maximum brightness) for each color to provide maximum temporal contrast of the stimulus.

### 2.2. Ocular Wavefront

The ocular wavefront was measured to characterize the optical quality of each participant’s eye and to explore whether the temporal resolution is affected by or related to the eye’s aberrations. A Laser Ray Tracing commercial device (iTrace, Tracey Technologies, Houston, TX, USA) was used for monocular wavefront measurements of the eye. Wavefront measurements were carried out with the same levels of light adaptation as the rest of the experiments. The parameters measured were the root mean square low- and high-order aberration (LOA and HOA RMS, respectively) values and the specific number of coma, spherical aberration, and trefoil HOA terms.

### 2.3. Photo-Stress Recovery Time

The photo-stress recovery time (PRT) test measures the macular function and is a potential biomarker for age-related macular degeneration disease. Therefore, recovery time after macular photobleaching may detect early functional deficits of cone photoreceptors [27]. A previously reported total disability glare vision optical instrument [28] was used to measure the time recovery after retinal photo-stress for a 100% Michelson Contrast visual target subtending 14° of the visual field.

### 2.4. Chromatic Critical Flicker Fusion Frequency

**Figure 2:**
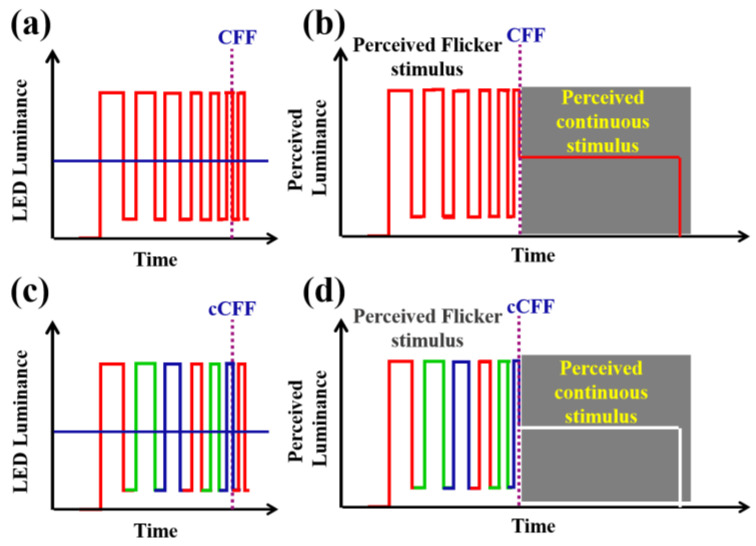
Illustration of Talbot’s law for monochromatic (**a**,**b**) and for trichromatic visual stimuli (**c**,**d**). Red, green and blue colors at the bottom images represent the trichromatic LED stimuli.

The Talbot–Plateau (T-P) law establishes a psychophysical aspect of vision related to temporal integration and visual persistence [29]. The T-P law states that when a flickering visual stimulus reaches the CFF, it will be perceived as a fused (continuous) stimulus with the same brightness as a steady stimulus with the same mean physical luminance [30]. In that sense, the concept of chromatic critical fusion frequency (cCFF) can be illustrated as shown in Figure 2. Figure 2a shows a periodically modulated luminance of a monochromatic light source with an increasing flickering rate. Figure 2b shows the luminance that an observer would perceive before reaching the CFF limit. Once the CFF threshold is exceeded, the visual perception is that of a continuous stimulus having the same luminance. Now let us consider the case of an RGB tristimulus pulse train (Figure 2c); the perceived luminance should be a steady stimulus of the average luminance of the sampled flicker. Then, if the red, green and blue channels merge, the visual perception of a trichromatic flicker stimulus beyond the cCFF limit should be a steady white visual perception (Figure 2d).

The experimental measurements of CFFs depend on physical factors such as brightness of the stimuli, wavelength, contrast, size, eccentricity [31] or individual patient characteristics [32]. Therefore, to establish reasonable comparisons, they must be under the same experimental conditions. The luminous intensity, viewing distance and ambient illumination are described in Table 2. The CFF measurements were carried out at the maximum nominal luminous intensity of the LED. For each subject, the perceived luminance depends on the conversion of luminous efficacy at the retina to the radiant flux entering the pupil of the eye.

### 2.5. Participants and Experimental Protocol

Thirty young and healthy European Caucasian volunteers (19 ± 1 years old) participated in this study, of which twenty were female and ten were males. None of them presented brain or cognitive disorders that could alter the assessment of temporal vision. Each subject, after 10 min of light adaptation to the ambient illumination (200 Lux), was subjected to three sequential measurements: (1) the ocular wavefront; (2) chromatic critical flicker fusion frequency; and (3) photo-stress recovery time. All measurements were performed monocularly in the right eye while the left eye was patched.

**Figure 3:**
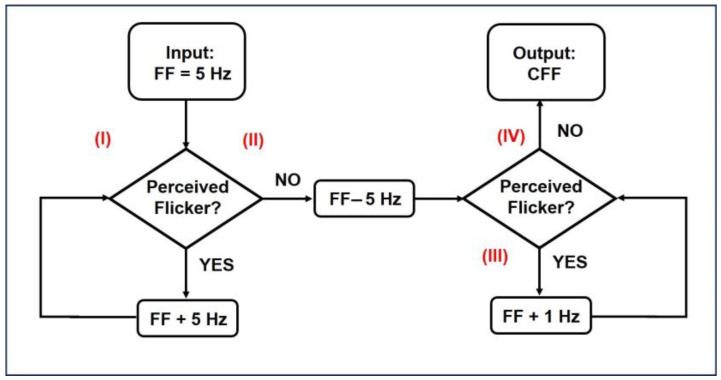
Flowchart describing CFF measurement protocol.

The cCFF experiments were carried out at a viewing distance of 600 mm from the RGB LED. For red, green, blue and achromatic stimuli CFF measurements, the volunteers were asked to indicate when the flickering light became steady. Regarding the chromatic CFF, participants were also asked to indicate when the tristimulus disappeared and continuous white light was perceived as follows: #Step (I): The examiner set an initial flicker frequency (FF) of 5 Hz and increased the FF in steps of 5 Hz by manual adjustment of the potentiometer (P in Figure 1). The frequency steps were monitored in a serial monitor of the Arduino digital programming interface—for each frequency step, 5 s of visual stabilization was allowed, after which the participant was asked if they were still perceiving the flicker stimulus. If so (verbal response), the frequency step was increased by another 5 Hz. #Step (II): Upon exceeding the FF threshold (i.e., the CFF), the participant reported a steady perception of the stimulus, and then the FF was decreased again until the flicker perception was recovered. #Step (III): At this point, the FF was again increased in discrete steps of 1 Hz until the refined threshold value was again obtained. #Step (IV): This refinement step was repeated twice for each subject, and the final output CFF was the average of these two values. Figure 3 summarizes the measurement protocol carried out.

### 2.6. Statistical Analysis

The statistical analysis initially consisted of performing the Shapiro–Wilk test to check the normality of the data for achromatic, red, green, blue and chromatic CCF data groups. Once the normality test revealed normally distributed experimental data, the One-Way Repeated Measures Analysis of Variance (One-Way RM ANOVA) test with Bonferroni correction explored for differences in the mean values among the treatment conditions. In addition, Equal Variance and F-Statistic Tests were performed to show whether the data were collected from populations with the same variance and to check whether the variability of the samples is greater than that expected from a random population variability. Finally, the effect sizes were computed as Cohen’s d.

On the other hand, the correlations of CFF with recovery time and ocular wavefront data consisted of Pearson Product Moment Correlation to explore possible relationships between the studied variables. The statistical analysis and graphical representation of data were carried out using Sigmaplot 12.0 scientific software. The Arduino circuit designs were obtained using a JavaScript AVR8js implementation available in [33].

## 3. Results

### 3.1. Chromatic Critical Flicker Fusion Frequency

**Figure 4:**
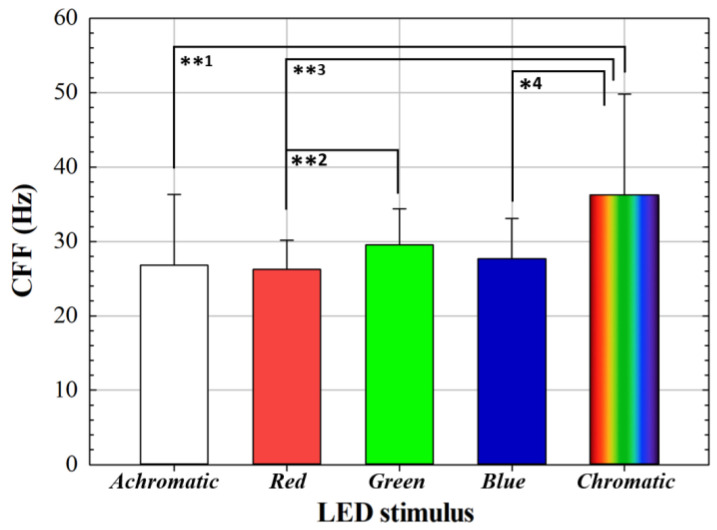
Mean CFF values for achromatic, red, green and blue colors and chromatic stimuli (lattice bar). The asterisks indicate those groups for which significant differences were found.

The RGB LED color can be easily modulated by modifying the Arduino Sketch, providing single color flickering for monochromatic CFF evaluation. Figure 4 compares the averaged CFF values for the achromatic stimulus (CFF = 26.79 ± 9.50 Hz) and for red (CFF = 26.26 ± 3.89 Hz), green (CFF = 29.56 ± 4.83 Hz) and blue (CFF = 27.68 ± 5.44 Hz) colors. The maximum CCF value was found for green light, which was found to be statistically higher than CFF for red light.

Furthermore, Figure 4 compares the cCFF value with those CFF values obtained for achromatic, red, green and blue colors. For a flicker frequency higher than 35.76 ± 14.03 Hz, the trichromatic flickering is perceived as steady white perception. The statistical analysis revealed that the critical frequency for the perception of fused white light is significantly higher than the CFF values for achromatic, red and blue separated colors, but not for the green stimulus. In addition, the Pearson correlation coefficient did not reveal any correlation between cCFF and the CFF for achromatic and red, green and blue colors. The statistical details of the results reported for Figure 4 are shown in Table 3. The average value of Cohen´s d was 0.795, which corresponds to a large effect size according to the classification reported in [34]. These statistical results reinforce the significance found between the CFF values as a function of the chromatic stimulus.

### 3.2. cCFF and Photo-Stress Recovery Time

The above results showed that cCFF is statistically longer than CFF for red and blue stimuli and achromatic light, but cCFF and the mentioned CFFs did not show any statistical relationships (Pearson Product Moment Correlation). Furthermore, the CFF for green light was found to be statistically greater than for the red stimulus. This section explores whether cCFF is related to the photo-stress recovery time (PRT).

The parvocellular pathway is sensitive to the red/green opponent channel in color vision [35], to high frequencies in spatial vision and to low-frequency stimuli in temporal vision [36], whereas the temporal processes are mediated in the magnocellular pathway. In addition, the red/green discrimination threshold is influenced by the macular pigment [37]. Then, considering the potential of the differential color flicker to detect color vision impairment [24], this work proposes the temporal red/green modulation contrast as a new parameter to explore the spatiotemporal sensitivity of vision. The red/green contrast modulation is defined as

(1) $$R/G\;Contrast\;Modulation=\frac{{CFF}_{G}-{CFF}_{R}}{{CFF}_{G}+{CFF}_{R}}$$

where CFF_G_ and CFF_R_ are the CFF values for green and red stimuli, respectively.

**Figure 5:**
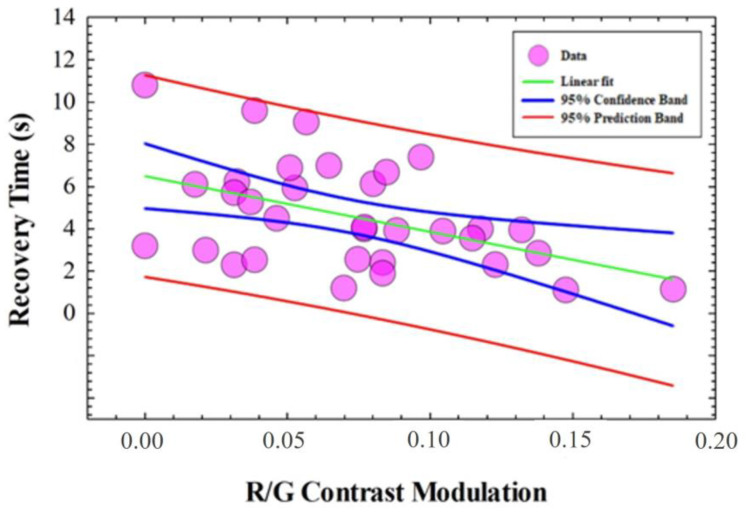
Recovery time as a function of the R/G contrast modulation. The green line and blue and red bands correspond to the best linear fit, confident and prediction bands, respectively.

A Pearson Product Moment Correlation was performed to establish any correlation between PRT and the cCFFs shown in Figure 4. Table 4 shows the results of the statistical analysis that revealed no correlation (Pearson Product Moment Correlation) between PRT and any CFF. However, a negative linear correlation was found (R^2^ = −0.45, *p* = 0.008) between the PRT and the R/G contrast modulation parameter as depicted in Figure 5.

### 3.3. Influence of the Optical Quality of the Eye on cCFF

Finally, this section analyzes whether spatial factors of the optical pathway of the visual processing have any implications in the temporal integration of trichromatic visual stimuli. Table 5 shows the averaged aberrometric data from wavefront measurements for all subjects involved in this study.

**Figure 6:**
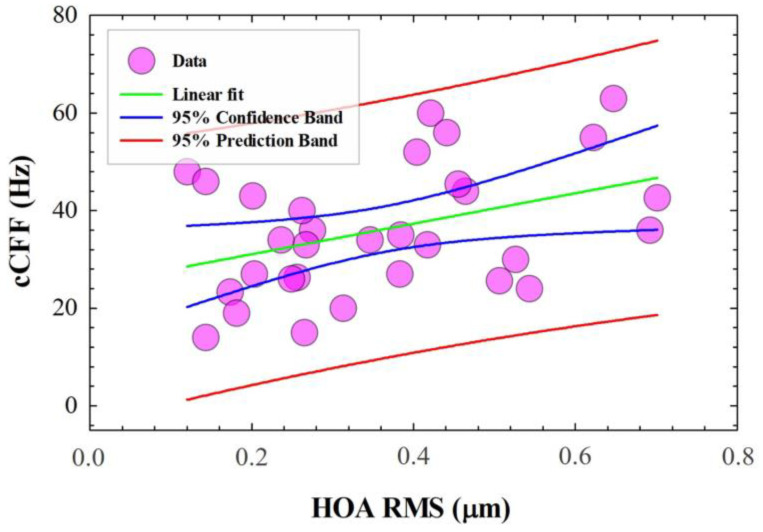
cCCF as a function of HOA RMS.

A statistical analysis revealed that cCFF is not affected by LOA (Pearson Product Moment Correlation, *p* = 0.426). However, a positive correlation was found between HOA and cCFF (R^2^ = 0.503, *p* = 0.004) as shown in Figure 6. These results show that high-order aberrations improve the trichromatic temporal resolution of vision. Specifically, the HOA coma and trefoil are the predominant terms of the HOA RMS. No relationships were found between HOA and CFF for red, green, blue and achromatic stimuli and R/G contrast modulation.

## 4. Discussion and Conclusions

This study presents a new cost-effective portable mini-device for trichromatic temporal resolution assessment of human vision powered by Arduino technology. The use of pulse-width modulation (PWM) signals to control the brightness and flicker frequency of LEDs controlled by Arduino devices may raise questions about possible limitations with respect to the use of other technologies based on data acquisition cards (DAQs). However, previous works have reported an accurate irradiance output [26] and timing precision of the LEDs based on PWM signals [38]. Those precision tests demonstrated the reliability of using Arduino boards for psychophysical experiments. In contrast, cost-effective LED driving Arduino-based systems can be controlled using open-source programming environments. Additionally, Arduino devices are based on open hardware processors that can be modified by the user to extend the main capabilities of Arduino or to communicate multiple boards wirelessly.

Teiraki et al. [26] reported an LED-based visual stimulator driven by an open-source Arduino microcontroller. Among other interesting applications, they demonstrated an application to measure the density of ocular media in humans based on heterochromatic flicker photometry.

Here, a new setup demonstrates an application for assessing the temporal resolution of visual performance using a flickering trichromatic stimulus. CFF was measured for the individual red, green and blue channels and for white light. The highest CFF threshold for green light was found to be significantly higher than for red light.

Regarding the cCFF, the integration frequency was significantly higher than the rest of the color stimuli except for the green stimulus. That is, while the visual perception of flickering of a monochromatic stimulus would disappear, a trichromatic stimulus flickering at the same frequency would continue to be visible.

Solomon et al. [39] reported on the temporal chromatic properties of the receptive fields of ganglion cells in the retina; they found cone-opponent responses only in a given temporal range frequency.

This fact could explain the differences between cCFF and CFF for achromatic and monochromatic stimuli whereby the red–green opponent response that shows the parvocellular subcortical pathway in the central retina is expressed at higher temporal frequency than for the single cone response.

Considering the association between the human visual perception of high-frequency flicker stimuli and cortical mechanisms [40], exploring the human visual perception of the trichromatic flicker at higher temporal frequencies could help better understand the outcomes of psychophysical tasks and isolate those contributions from the magnocellular pathway.

Section 3.2 explored the relationships between the chromatic temporal resolution of the visual system and macular function as measured by photo-stress recovery time (PRT). The CFF reflects the temporal resolution of vision to which magnocellular cells are sensitive [41] while the PRT is related to macular pigment optical density [42] and spatial contrast sensitivity [43], so those parameters are not expected to be related due to the antagonistic processes of the parvo- and magnocellular pathways [44].

In that sense, no relationships were found between CFFs for red, green and blue light; cCFF; and PRT. However, a statistical negative correlation was found between the red–green modulation contrast and PRT. Theoretically, the presence of a macular pigment is to enhance the visual performance in glare vision conditions [45]. In that sense, a higher macular pigment density will result in shorter PRTs. The results reported here showed that PRT and cCFF are independent factors of the visual function. Therefore, the contrast modulation parameter defined for red and green CFF measurements appears to be related to the macular pigment density rather than temporal processing of color vision.

Finally, Section 3.3 studied the influence of ocular aberrations on the chromatic CFF. No relationships were found between cCFF and low-order aberrations (i.e., refractive errors); however, a positive linear correlation was found between HOA and cCFF. The results demonstrate that high-order ocular aberrations improve the temporal resolution of the visual function.

Spatial contrast sensitivity and HOA are inversely correlated; one of the main mechanisms that degrade visual quality in corneal diseases such as keratoconus is the presence of increased HOA [46]. Furthermore, HOA displays a compensatory mechanism in spatial contrast sensitivity when intraocular scattering effects are also significant [47]. However, the influence of ocular aberrations on temporal aspects of vision is lacking in the literature.

A possible explanation for the relationship between HOA and cCFF can be found in the spatiotemporal mechanisms in human vision processing: spatial and temporal information interact with the magnocellular and parvocellular pathways, respectively. More specifically, there is an inhibitory parvo-magnocellular interaction: improving temporal resolution deteriorates spatial resolution [48]. Therefore, it can be concluded that while on the one hand HOA degrades the optical quality of the eye (in terms of spatial resolution), on the other hand, it improves the temporal resolution.

Recently, it has been reported that the human visual cortex is sensitive to flicker stimuli that induce changes in neural activity [49]. In neurodegenerative diseases such as Alzheimer’s, the brain undergoes electrophysiological changes that are susceptible to reacting to neurostimulation therapies based on chromatic flickering at 40 Hz [50]. One of the drawbacks of studying brain activity with perceived flickering is a high level of patient discomfort [51]. The results reported here showed a mean value for cCFF of 37.76 ± 14.03 Hz; then, a trichromatic flicker stimulus at 40 Hz falls inside the steady perceived stimuli regime according to the Talbot´s law, allowing brain activity to be studied at that critical frequency while the patient looks at a continuous white visual stimulus.

To conclude, a new cost-effective mini-device for the study of the polychromatic temporal resolution of visual function based on Arduino technology is presented. The cCFF device allowed us to study the critical flicker fusion of a chromatic stimulus, providing a steady white visual perception beyond the critical frequency limit. The contrast modulation of CFF values for red and green stimuli was found to be related to photo-stress recovery time and therefore more related to macular pigment density than to the temporal resolution of color vision. Furthermore, increased ocular HOA improves the temporal resolution of the visual function.

Future work will include the characterization of the temporal sensitivity of color vision using the chromatic CFF device in patients with retinal visual impairment.

## Figures and Tables

**Table 1:** Arduino Sketch to control the cCFF device.

Arduino Sketch
int redPin = 9;
int redPin = 10;
int redPin = 10;
void setup () {
pinMode (redPin, OUTPUT);
pinMode (greenPin, OUTPUT);
pinMode (bluePin, OUTPUT);
Serial. Begin(9600);
}
void loop () {
int sensorValue (analogRead(A0));
Serial.println (sensorValue);
SetColor (0,0,0);
SetColor (255,0,0);
delay (analogRead(A0));
SetColor (0,0,0);
SetColor (0,255,0);
delay (analogRead(A0));
SetColor (0,0,0);
SetColor (0,0,255);
delay (analogRead(A0));
}
void SetColor (int Red, int Green, int Blue)
{
analogWrite (redPin, red);
analogWrite (greenPin, green);
analogWrite (bluePin, blue);

**Table 2:** Experimental conditions for the CFF measurements.

Parameter	Measure
Luminous intensity [red]	2600 mcd
Luminous intensity [green]	2000 mcd
Luminous intensity [blue]	1800 mcd
Viewing distance	600 mm
Ambient illumination	200 Lux
Eccentricity	Foveal vision
CFF red	CFF for red stimulus (Hz)
CFF green	CFF for green stimulus (Hz)
CFF blue	CFF for blue stimulus (Hz)
CFF achromatic	CFF for white light stimulus (Hz)
Chromatic CFF (cCFF)	CFF for mixed red, green and blue lights

**Table 3:** Equal Variance test (EVT), F-Statistics Test (F), *p* value and Cohen´s d outputs of the statistical analysis carried out in Figure 4.

Compared Stimuli	EVT	F	*p*	Cohen’s d
Achromatic vs. chromatic	1	10.84	0.004	0.75
Red vs. green	1	10.31	0.002	0.75
Red vs. chromatic	1	15.74	0.003	0.92
Blue vs. chromatic	1	9.46	0.021	0.76

**Table 4:** Correlation results (*p* value) of the PRT versus achromatic, red, green, blue and trichromatic (RGB) stimuli, and R/G contrast modulation.

	Achromatic	RED	GREEN	BLUE	RGB	R/G
PRT	*p* = 0.836	*p* = 0.812	*p* = 0.506	*p* = 0.772	*p* = 0.731	*p* = 0.008

**Table 5:** Root mean square for low- (LOA RMS) and high-order aberrations (HOA RMS), and individual contribution of the coma, spherical aberration (SA) and trefoil high-order terms.

LOA RMS	HOA RMS	Coma	SA	Trefoil
2.12 ± 2.52 μm	0.36 ± 0.17 μm	0.21 ± 0.14 μm	0.10 ± 0.07 μm	0.18 ± 0.11 μm

## Data Availability

Dataset is available upon reasonable request.
